# Responses of Ecosystem CO_**2**_ Fluxes to Short-Term Experimental Warming and Nitrogen Enrichment in an Alpine Meadow, Northern Tibet Plateau

**DOI:** 10.1155/2013/415318

**Published:** 2013-12-29

**Authors:** Ning Zong, Peili Shi, Jing Jiang, Minghua Song, Dingpeng Xiong, Weiling Ma, Gang Fu, Xianzhou Zhang, Zhenxi Shen

**Affiliations:** ^1^Lhasa National Ecological Research Station, Key Laboratory of Ecosystem Network Observation and Modelling, Institute of Geographic Sciences and Natural Resources Research, Chinese Academy of Sciences, A11 Datun Road, Chaoyang District, Beijing 100101, China; ^2^University of Chinese Academy of Sciences, No. 19A Yuquan Road, Shijingshan District, Beijing 100049, China; ^3^Key Laboratory of Ecosystem Network Observation and Modelling, Institute of Geographic Sciences and Natural Resources Research, Chinese Academy of Sciences, A11 Datun Road, Chaoyang District, Beijing 100101, China

## Abstract

Over the past decades, the Tibetan Plateau has experienced pronounced warming, yet the extent to which warming will affect alpine ecosystems depends on how warming interacts with other influential global change factors, such as nitrogen (N) deposition. A long-term warming and N manipulation experiment was established to investigate the interactive effects of warming and N deposition on alpine meadow. Open-top chambers were used to simulate warming. N addition, warming, N addition × warming, and a control were set up. In OTCs, daytime air and soil temperature were warmed by 2.0°C and 1.6°C above ambient conditions, but soil moisture was decreased by 4.95 m^3^ m^−3^. N addition enhanced ecosystem respiration (Reco); nevertheless, warming significantly decreased Reco. The decline of Reco resulting from warming was cancelled out by N addition in late growing season. Our results suggested that N addition enhanced Reco by increasing soil N availability and plant production, whereas warming decreased Reco through lowering soil moisture, soil N supply potential, and suppression of plant activity. Furthermore, season-specific responses of Reco indicated that warming and N deposition caused by future global change may have complicated influence on carbon cycles in alpine ecosystems.

## 1. Introduction

Over the past 50 years, the Tibetan Plateau has experienced pronounced warming [1–3]. By the end of 21st century, the magnitude of mean global temperature is projected to increase by 1.8 to 4.0°C and alpine regions are believed to be exposed to an even higher rate of warming than mean global level [2, 4]. Moreover, alpine ecosystems might be more sensitive because plant growth is often accustomed to low temperature environment [5], and soil respiration is more sensitive to warming at lower temperature [6]. In addition to the direct effects of climate warming on ecosystem productivity due to an extended growing season, warming may alter productivity indirectly by influencing soil N dynamics [7]. Increased rates of N mineralization driven by climate warming may improve plant N availability and could stimulate plant growth in N-limited systems, provided water is not limiting [8, 9]. However, increased N mineralization over winter at a time when plant roots are largely inactive, coupled with an increased frequency of soil freeze-thaw cycles, may increase the possibility of soil N leaching losses and thus affect plant productivity and soil C dynamic in the following growing season due to N limitations of alpine ecosystems [10, 11]. Better understanding of the response of ecosystem CO_2_ flux to global warming, increasing nitrogen deposition, and their interactions is crucial to project carbon cycling in the future global change scenarios.

The extent to which increased warming will drive changes in plant productivity and ecosystem C flux over the next century will depend on how climate warming interacts with other influential global change factors, such as N deposition, to affect the N retention of ecosystems [12, 13]. Generally, N deposition can increase productivity and biomass accumulation in the short term [14] because alpine ecosystems are primarily N limited caused by low temperature [15, 16]. In addition, N fertilization has led to small, initial increases in soil CO_2_ efflux but has generally suppressed it in chronically amended plots [17–19]. Likewise, N additions have initially increased N mineralization, but elevated rates of N cycling have either returned to control levels [20, 21] or declined in the long run [22]. Thus when climate warming and N deposition occur separately, they appear to have opposite effects on soil C and N dynamics, with warming causing long-term soil C losses and high N turnover, but N deposition resulting in soil C gain and low rates of N cycling. However, few studies have explored the interactive effects of climate warming and N deposition on plant productivity and ecosystem C dynamics, especially in N-limited alpine ecosystems.

Ecosystem respiration is primarily controlled by soil temperature and soil water content and complex interactions between them [23]. In semiarid and arid regions, soil water content is the main factor regulating plant growth and soil microbial activity [24–26] and subsequently mediates the apparent temperature sensitivity to soil temperature [27, 28]. Besides the direct effect on ecosystem respiration by elevating temperature, experimental warming can also indirectly decrease ecosystem CO_2_ fluxes by altering (mostly reducing) soil water availability, especially in months with few precipitation events in semiarid and arid regions.

In this study, we examined the interactive effects of simulated climate warming and N deposition on ecosystem CO_2_ efflux, soil N dynamic, and plant productivity in a semiarid alpine meadow on the Tibetan Plateau. We conducted year-round warming treatments by open-top chambers (OTCs) crossed with N enrichment treatments (4 g N m^−2^ year^−1^) in a factorial design. We hypothesize that warming would decrease plant productivity and ecosystem CO_2_ efflux, caused by indirect decrease of soil moisture in this semiarid region. We also predicted that N enrichment would counteract the effects caused by simulated warming through increase of water and N availability in late growing season.

## 2. Materials and Methods

### 2.1. Site Description

This study was carried out in the grassland station of Damxung County (91°05′ E, 30°51′ N, 4333 m a.s.l) in the south-facing slope of Nyainqentanglha Mountains, north Tibetan Plateau. A detailed site description was introduced in literature [16, 29]. According to observations from 1963 to 2010 at Damxung meteorological station (4288 m a.s.l, ca. 3 km away from our experimental plot), annual mean air temperature increased by 1.6°C while precipitation showed a declining trend from 1963 to 1990 but an increasing trend from 1991 to 2010 [30].

### 2.2. Experimental Design and Microclimate Monitoring

The field manipulations consisting of warming (year-round warming and control) crossed with two N addition treatments (added N and control) were organized in a randomized block design with five replicates for each of the four treatments. We followed methods of the International Tundra Experiment and applied ten open-top chambers (OTCs), the passive warming device to generate artificially warmed conditions [2, 31] for five controls and five N addition plots. The OTCs, with 100 cm diameter of top opening, 140 cm diameter of bottom, 40 cm in height, and a bottom area of 1.54 m^2^, are made of 3 mm thick polycarbonate plastic. This material has high solar transmittance in visible and ultraviolet wavelengths (about 90%) [32]. In N added plots, we applied a pulse of aqueous ammonium nitrate (NH_4_NO_3_) at a rate of 2 g N m^−2^ year^−1^ in early June and early August, respectively. These addition rates are designed to approximate projected increases in atmospheric deposition in this region by the year of 2050 [33]. We set up the warming plots in early July 2010 and synchronously monitored the warming effects on year-round air temperature, soil moisture, and temperature at 5 cm depth by a HOBO weather station (Onset Inc., Bourne, MA, USA) on half-hour frequency. The distance for buffer between each replicate was at least 3 m.

### 2.3. Measurement of Aboveground and Belowground Biomass

A nondestructive sampling method was used to estimate aboveground biomass [34–36]. Briefly, the average height and cover of vegetation canopy were measured using a 50 cm × 50 cm quadrat divided into twenty-five 5 cm × 5 cm subsquares in each plot on July 01, July 25, August 25, and September 17 in 2012. We also carried out this process in nearby alpine meadow on each sampling date by measuring mean height and cover of vegetation canopy, harvesting, oven-drying, and weighing the vegetation materials. The equation that was used to simulate the relationship between aboveground biomass (AGB) and vegetation height (H) and cover (C) is AGB = 0.269 + 3.466 C + 0.752 H (*R* ^2^ = 0.658, *P* < 0.001, and *N* = 80). We also used a soil-drill sampler (5 cm in diameter) to take 0~10 cm and 10~20 cm soil samples in mid-August and these root samples were immediately washed, separated, oven-dried at 65°C for 48 h, and weighed.

### 2.4. Soil Net N Mineralization

Soil net N mineralization was measured using the buried soil core technique employed by [37, 38]. The concentrations of extractable NO_3_^−^-N and NH_4_^+^-N were compared in initial and incubated soil cores in situ for approximately three weeks. While taking one soil sample core for immediate analysis, another three intact soil cores were taken out, placed into a polyvinyl chloride (PVC) collar (5 cm in diameter and 12 cm in height) at a depth of 10 cm, resituated into the soil, and sealed with plastic wraps which can prevent N deposition by rainfall and maintain enough ventilation [39]. Every three weeks after soil sampling, the incubated cores were taken out from soil holes on July 25, August 16, and September 6, 2012, respectively. Soils in the cores were immediately passed through a 2 mm sieve to remove roots, gravel, and stones. Within 48 hours from each soil sampling, NO_3_^−^-N and NH_4_^+^-N of initial and incubated cores were extracted using 2 mol L^−1^ KCl and filtered and analyzed on a continuous flow analyzer (AA3, SEAL Analytical, Germany). The differences of inorganic N (mg kg^−1^, total summary of NO_3_^−^-N and NH_4_^+^-N) between the initial and incubated soil cores divided by incubated time (day) were used to estimate the rate of inorganic N mineralization (mg kg^−1^ d^−1^).

### 2.5. Measurement of Ecosystem Respiration

During the growing season, ecosystem respiration was measured from June to September in 2012 by a portable soil CO_2_ flux system (LI-8100, LI-COR Biosciences, Lincoln, NE, USA). Briefly, PVC chambers with 20 cm in diameter and 5 cm in height were inserted into the soil to a depth of 3 cm one month before our measuring process and intact plant was kept in PVC chambers. Ecosystem respiration was measured at 3-hour intervals in July 22~23 and August 21~22 from 18:00 p.m. to 18:00 p.m. (local time) in the next day to achieve the diurnal variation pattern. Nine cycles of data were obtained in each measurement date. In addition, CO_2_ fluxes between 09:00 and 11:00 a.m. (local time) were also measured every 10 days to represent daily mean flux because the CO_2_ fluxes in these periods are equal to daily averages according to previous studies in this site in the growing season [16, 40]. In all, ten daily average CO_2_ fluxes were obtained throughout the growing season.

### 2.6. Statistical Analysis

To examine the effects of warming on temperature sensitivity of ecosystem CO_2_ flux, exponential regression models *R* = *a*e^*bT*^ were used, in which *R* is the ecosystem CO_2_ flux, *T* is the soil or air temperature, coefficient *a* is the ecosystem CO_2_ flux when temperature is zero, and coefficient *b* represents the temperature sensitivity of ecosystem CO_2_ flux. *Q*_10_ value, calculated as *R*_*T*+10_/*R*_*T*_, where *R*_*T*_ and *R*_*T*+10_ are ecosystem CO_2_ fluxes at temperature *T* and *T* + 10, respectively, was used to evaluate the dependence of ecosystem CO_2_ flux on temperature on diurnal scales. Regression analyses were performed to test the dependence of ecosystem CO_2_ flux on soil water content, soil temperature, plant aboveground biomass, and soil inorganic N content at seasonal scales. Repeated-measure ANOVA was applied to assess the effects of warming and N addition on ecosystem CO_2_ flux. One-way ANOVA analyses followed by Tukey multiple comparison were used to examine the differences in monthly average ecosystem CO_2_ flux, plant biomass, soil inorganic N content and net mineralization rate among treatments during growing season. Before each analysis, all data were tested for homogeneity. If not, they were ln-transformed before analysis. Significance level was 95% confidence interval. All the analyses were performed in SPSS 16.0 (SPSS for Windows, Version 16.0, Chicago, USA).

## 3. Results

### 3.1. Warming Effects on Microclimate

**Figure 1:**
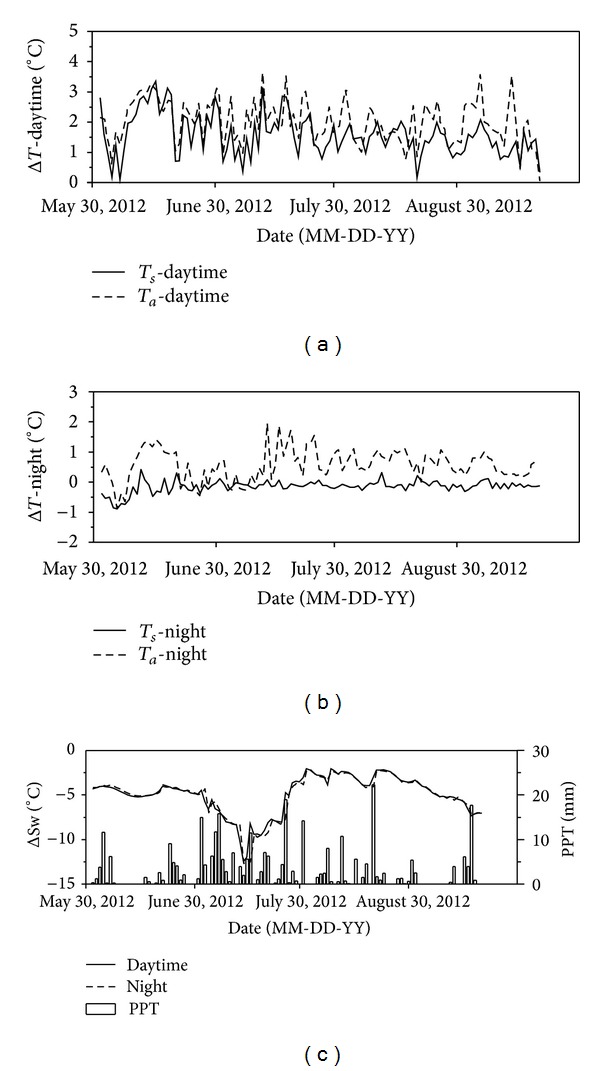
Effects of simulated warming on meteorological factors over growing season in 2012. Daytime and nighttime air temperature (*T*_*a*_), soil temperature (*T*_*s*_), and soil water content (Sw) at the depth of 0~5 cm represent the difference between the values inside OTCs and the ambient conditions in daytime (07:30~20:30) and nighttime (00:00~7:30 and 20:30~24:00), respectively. PPT represents the daily precipitation from June 1 to September 30, 2012.

Simulated warming significantly resulted in an average increase of 2.0°C daytime air and 1.6°C soil temperature compared with ambient conditions during growing season (from June to September; Figure 1(a); *P* < 0.05). However, warming was not significant in nighttime air temperature and increased by only 0.6°C in soil temperature (Figure 1(b); *P* > 0.05). On the contrary, soil water content in day and night was indiscriminately decreased by 4.95 m^3^ m^−3^ resulting from simulated warming (Figure 1(c)), causing a warmer and dryer condition in the OTCs. Correlation analysis showed that the decrease of soil water availability during daytime was marginally correlative with the increase of soil temperature (*P* = 0.096). Precipitation was 312.7 mm during growing season in 2012, mainly concentrating in July and August (49% and 24% of the total, resp.) (Figure 1(c)).

### 3.2. Diurnal and Seasonal Variations of Ecosystem Respiration

**Figure 2:**
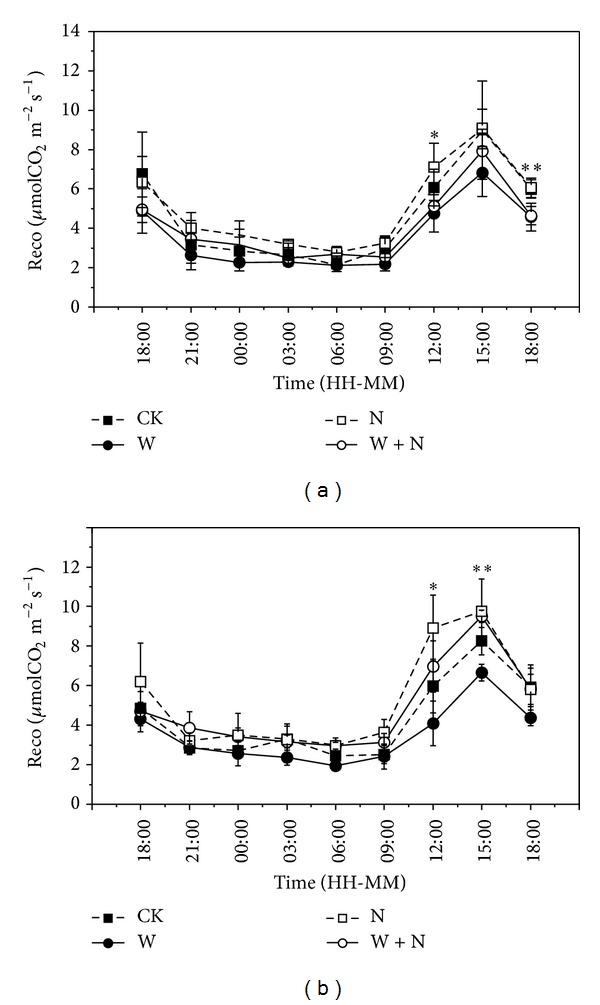
Diurnal variations of soil respiration under different treatments in alpine meadow measured on July 22~23 (a) and August 21~22 (b), 2012. CK, control (solid square with dot line); N, N addition (open triangle with dot line); W, warming (solid square with straight line); W + N, warming plus N addition (open triangle with straight line). Values represent the mean ± SE (*n* = 4).

**Figure 3:**
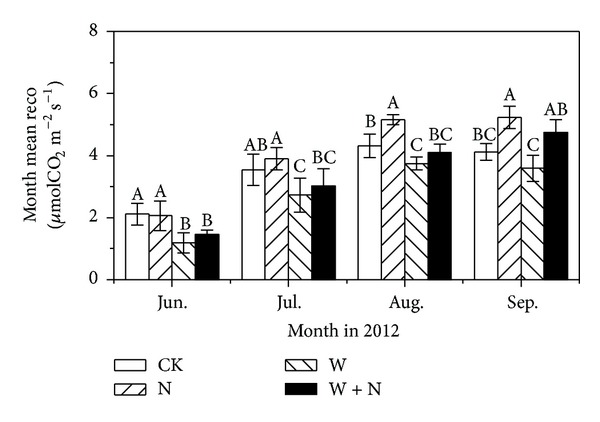
Monthly mean soil respiration over growing season under different treatments in alpine meadow in 2012. See Figure 2 for the abbreviations of treatments.

Ecosystem CO_2_ fluxes showed diurnal variations with peak values at 15:00 p.m. and minimum values at 03:00~06:00 a.m. in two measuring dates in growing season (Figure 2). Moreover, ecosystem CO_2_ fluxes also showed seasonal variations with peak values in mid-August (Figure 3).

N addition had no effects on ecosystem CO_2_ fluxes in June and July but significantly increased them by 16% and 21% in August and September (Figures 2 and 3; Table 1; *P* < 0.05), respectively. Warming significantly decreased ecosystem CO_2_ fluxes by 44% and 23% in June and July (Figure 3; *P* < 0.05), respectively, while the effects were not pronounced in September (Figure 3; *P* > 0.05). N addition and warming significantly decreased ecosystem CO_2_ fluxes by 31% in June (Figure 3; *P* < 0.05), but the effects were not pronounced compared with control treatment in the following period, indicating that N enrichment cancelled out the decline of warming effects on ecosystem CO_2_ fluxes in peak growing season.

### 3.3. Plant Production

**Figure 4:**
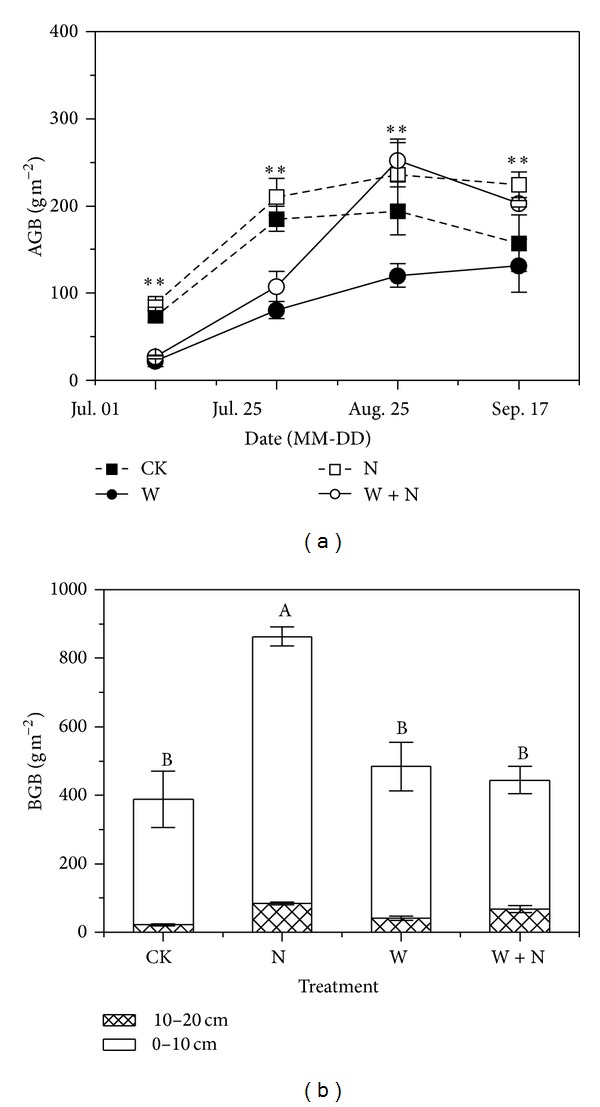
Seasonal variations of plant aboveground and belowground biomass over growing season under different treatments in alpine meadow in 2012. See Figure 2 for the abbreviations of treatments.

N addition significantly increased aboveground and belowground biomass (Figure 4). For example, N addition increased aboveground biomass by 18% (Figure 4(a); Table 1; *P* < 0.05) and belowground biomass by 55% (Figure 4(b); *P* < 0.01), respectively, in August. On the contrary, warming significantly decreased aboveground biomass by 38% (Figure 4(a); Table 1; *P* < 0.05) in August. N addition and warming treatments decreased aboveground biomass in early growing season but this trend reversed in late growing season (Figure 4(a)). For example, aboveground biomass decreased by 41% (*P* < 0.05) in July while increasing by 29% (Figure 4(a); *P* < 0.05) in September under N addition and warming treatments.

### 3.4. Soil Inorganic N Content and Mineralization Rate

**Figure 5:**
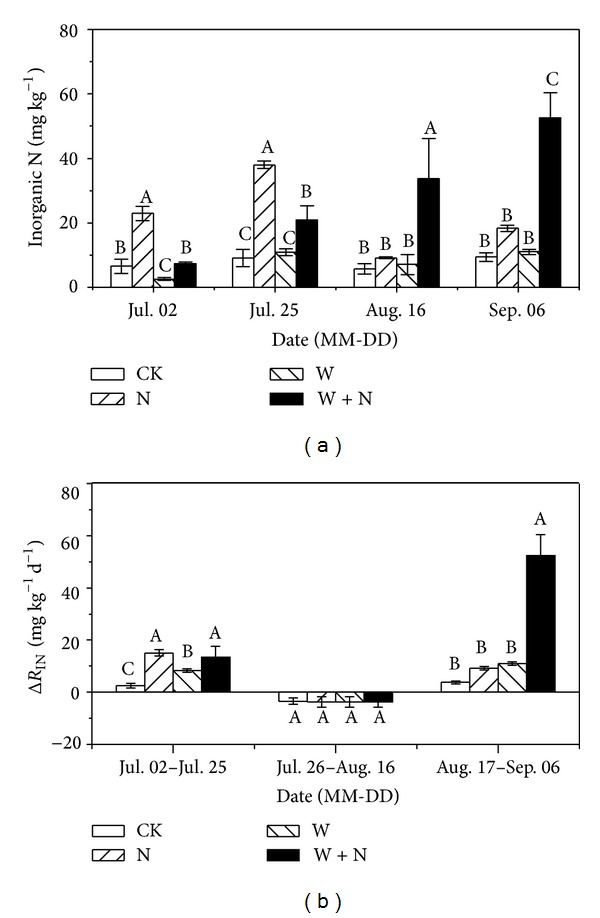
Seasonal variations of soil inorganic N (a) and soil net N mineralization (b) over growing season under different treatments in alpine meadow in 2012. See Figure 2 for the abbreviations of treatments.

N addition enhanced soil mineral N content and mineralization rate in early growing season (Figure 5, *P* < 0.05) while warming decreased soil mineral N content in this season. In middle growing season, the rate of soil microbial immobilization was higher than mineralization rate (Figure 5(b)). However, N addition combined with warming significantly increased soil mineral N content by 4.5 times and mineralization rate by 12.9 times at the end of growing season (Figure 5, *P* < 0.01).

### 3.5. Controlling Factors

**Figure 6:**
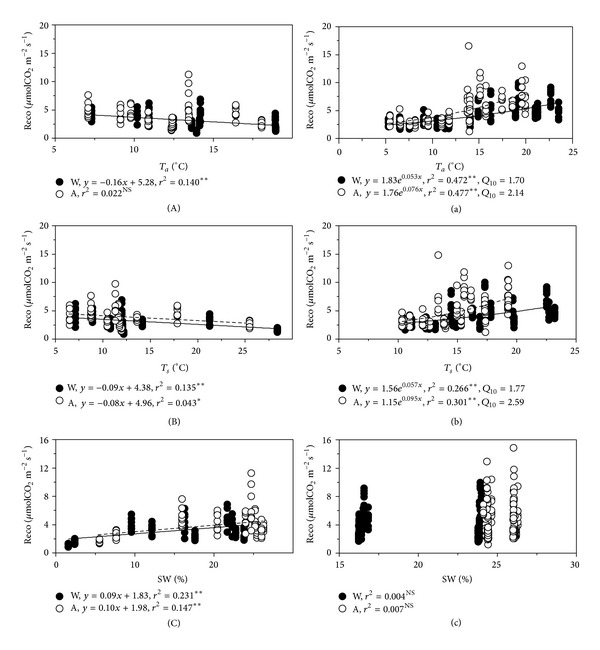
Dependence of soil respiration on air temperature (A, a), soil temperature (B, b), and soil water content (C, c) on seasonal (A, B, C) and diurnal (a, b, c) scales. Solid circles represent simulated warming treatment (W) and open circles represent ambient treatment (A). NS, ∗, and ∗∗ represent significant levels *P* ≥ 0.05, *P* < 0.05, and *P* < 0.01, respectively. The following is the same. *Q*_10_ represents the sensitivity of ecosystem respiration to temperature.

Linear regression analysis showed that seasonal variations of ecosystem CO_2_ flux were negatively correlated with air temperature and soil temperature, whereas ecosystem CO_2_ flux was positively correlated with soil water content at the depth of 5 cm (Figures 6(A)~(C)), especially in warming treatments, indicating that decline of soil water content and increase of soil and air temperature in warming treatments accounted for the decline of ecosystem CO_2_ flux at seasonal scale. However, diurnal variations of ecosystem CO_2_ flux were exponentially correlated with air temperature and soil temperature. Air temperature and soil temperature could explain 47.2%~47.7% and 26.6%~30.1% of its diurnal variations, respectively. We also found that warming treatments decreased the temperature sensitivity (*Q*_10_) of CO_2_ fluxes, which was 1.70 response to air temperature and 1.77 to soil temperature in warming treatments compared with 2.14 to air temperature and 2.59 to soil temperature (Figure 6, *P* < 0.05), respectively.

**Figure 7:**
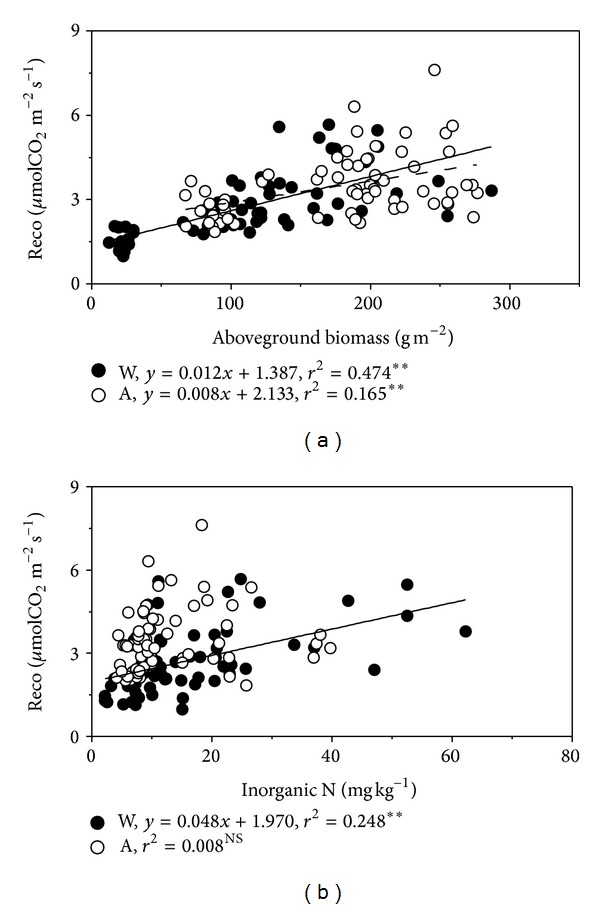
Dependence of soil respiration on aboveground biomass (a) and inorganic N content (b). Solid circles represent simulated warming treatment (W) and open circles represent ambient treatment (a). Regression lines are indicated only for significant correlations, with solid line and dash line for warming and ambient treatment, respectively.

Ecosystem CO_2_ fluxes exhibited significant correlation with aboveground biomass and soil inorganic N content (Figure 7), indicating that plant production and soil N availability were critical factors regulating seasonal variations of ecosystem CO_2_ fluxes. More pronounced above-mentioned correlations were found in warming plots than the ambient plots, illustrating that the decline of biomass and soil N availability resulting from warming accounted for the decrease of ecosystem CO_2_ fluxes in warming plots.

## 4. Discussion

Our results showed that short-term N addition enhanced plant biomass and ecosystem CO_2_ flux, but the increase of CO_2_ flux was significant only in late growing season. On the contrary, warming decreased CO_2_ flux and plant biomass over the whole growing season, with most pronounced effects in June and July. N addition offset the decrease of soil respiration and plant biomass resulting from warming. The above results corroborate our hypotheses and highlight the dampening effects of reduced soil water conditions on N availability and ecosystem CO_2_ fluxes caused by indirect effect of warming and additive effects of nitrogen and warming in late growing season.

### 4.1. Warming Effects

Warming significantly reduced aboveground biomass (Figure 4) and ecosystem respiration (Figure 3), and these effects were pronounced in early growing season. Generally, warming may increase plant N availability by enhancing rates of N mineralization, which, coupled with an extended growing season, could promote plant growth in N-limited ecosystems, provided soil water is not limiting [8, 9]. However, early growing season in present study site is the time that precipitation events are generally rare. The reduction of soil water content in warming treatments suppressed the potentially positive effects of warming on plant growth and C fluxes. In addition, warming over nongrowing season, combined with microbial C limitation at this time [41, 42], could enhance N mineralization. But plant activities are largely dormant and soil microorganisms are mostly absent for N immobilization; thus winter warming may cause large amount of N leaching or trace gases emission during increased frequency of soil freeze-thaw cycles [43–45]. In our study, soil mineral N content in warming plots was very low in early growing season possibly due to large loss of N during winter warming (Figure 5). This may render negative effect on plant growth. As plant N uptake in early growing season was of great importance for plant productivity in the following season due to N limitations of alpine ecosystems [46], thus, warming decreased ecosystem CO_2_ fluxes through the suppression of production productivity as ecosystem respiration in growing season may primarily depend on the utility of recent plant photosynthates [37, 47].

In the growing season, the passive warming by OTCs significantly enhanced daytime soil and air temperature while decreasing soil water content (Figure 1), which may have great impacts on plant community and CO_2_ efflux in early growing season when rainfall events are rare. In semiarid region, soil moisture is a key environmental factor controlling season variations [16] and large-scale pattern [48] of ecosystem CO_2_ flux. The decline of soil water availability caused by warming directly suppressed ecosystem CO_2_ fluxes. In addition, soil microbial carbon and N were also reduced by warming because warming may inhibit the activities of soil microorganisms and substrate supply for its reproduction due to the decline of soil water availability [2, 49]. In addition, the decline of soil N availability in OTCs also accounted for the decrease of ecosystem CO_2_ fluxes, as N availability in soil may directly affect soil microbial activities.

### 4.2. Nitrogen Enrichment Effects

N enrichment treatment significantly enhanced above- and belowground biomass in peak and late growing seasons (Figure 4), accompanied with stimulation of ecosystem CO_2_ fluxes (Figure 3). Generally, soil N mineralization is very slow due to high altitude and low temperature on the Tibetan Plateau, leading to the limitation of N availability on plant production [16, 50]. Exogenous N input could speedily enhance plant N availability in soil, resulting in the increase of N content in plant leaves and promotion of photosynthetic capacity [51]. Our results showed that N enrichment also accelerated soil net N mineralization in early growing season (Figure 5), which could stimulate the decomposition of soil organic matter [52] and in turn increase soil N content and subsequently promote plant production [38]. Increased plant productivity means more plant growth and maintenance respiration [23] and more plant photosynthates supply to soil microorganisms [26, 53]. Therefore, the increase of plant productivity caused by N enrichment was an important source for the increase of ecosystem CO_2_ fluxes.

Plant aboveground biomass in N addition combined with warming treatment was significantly higher than that in warming treatments (Figure 4), indicating that exogenous N input compensated the effects of low soil N content and soil water availability on plant production and soil microbial activities caused by warming, especially in late growing seasons (Figure 5). In line with another study in a temperate old field during exceptionally dry summer [54], plant aboveground biomass under N addition treatments had no significant difference with that under N addition combined with warming treatments in this semiarid alpine ecosystem (Figure 4), demonstrating that precipitation distribution appears to play a key role in modulating the effects of climate warming and enhanced N deposition [54].

### 4.3. Factors Regulating Ecosystem CO_2_ Flux

Soil temperature is the main environmental factor driving changes in respiration rate [55]. The positive relationship between CO_2_ flux and soil temperature has been included in many models related to carbon cycling [56, 57]. Our results demonstrated that soil and air temperature were critical factors that exponentially correlated with ecosystem respiration which could explain 26.6% and 47.2% of diel variations of ecosystem respiration under warming treatments and 30.1% and 47.7% in ambient treatments (*P* < 0.01). We found temperature sensitivity of CO_2_ efflux (*Q*_10_) by Van't Hoff's equation [58] was decreased with simulated warming, which is coincident with other researches [59], suggesting that plant growth and diurnal variations of ecosystem CO_2_ fluxes in high altitude and cold-climate ecosystems are mainly limited by low temperature [2]. On the contrary, at the seasonal scales it was negatively correlated with air and soil temperature but positively correlated with soil moisture (Figure 6) because the soil water availability plays decisive role in affecting plant productivity [24–26], soil microbial activity [60, 61], and consequently the response of ecosystem CO_2_ flux to temperature [62] in this semiarid region. In addition, along with plant growth, photosynthetic capacity increases and supplies more substrates to belowground biological processes, which is confirmed by positive correlation between ecosystem CO_2_ flux and aboveground biomass (Figure 7(a)). Thus the relationship between ecosystem CO_2_ flux and temperature was confounded by soil water availability and plant productivity, which is consistent with another study results in this area [16]. Besides, ecosystem CO_2_ flux was positively correlated with soil inorganic N content under warming treatments (*P* < 0.01) but not in ambient conditions (Figure 7(b); *P* > 0.05), indicating that the decrease of soil inorganic N content under simulated warming treatments imposed restrictions on soil respiration.

## 5. Conclusions

The present study revealed that short-term N addition enhanced ecosystem CO_2_ flux by increasing soil N availability and plant production, whereas warming decreased it through reducing soil moisture and soil N supply and subsequently suppressed plant activities and N enrichment could make up for this reduction to some extent. In addition, we found that the alteration of soil water content caused by warming mediated the warming effects on fate of ecosystem C and N cycling. Warming in this semiarid alpine meadow ecosystem has consequences for soil N cycles and ecosystem C fluxes that differentiate from other ecosystems.

**Figure 8:**
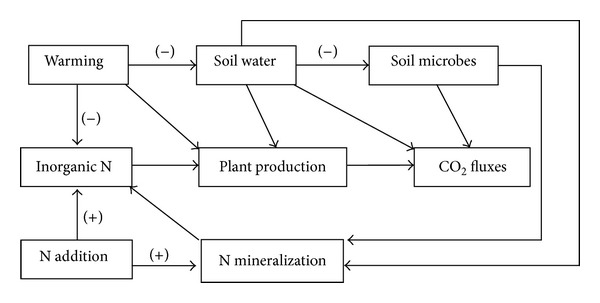
Conceptual diagram of experimental warming and N enrichment on ecosystem C and N cycling in semiarid alpine meadow. “+” refers to positive stimulation effect and “−” refers to inhibiting effect.

Based on our results, we propose a conceptual diagram of how experimental warming and N addition affect C and N cycling in semiarid alpine meadow ecosystem (Figure 8). Besides the direct effect on plant production, warming alters plant production by decreasing soil water content and increasing inorganic N loss, especially in early growing season when precipitation is rare. The reduction of soil water availability can directly suppress ecosystem CO_2_ fluxes and inhibit soil microbial activities and indirectly affect ecosystem C cycling. Furthermore, the decrease of soil water content may have influences on N mineralization and subsequently alter the N supply to plant. Increased precipitation in late growing season has the potential to relieve soil water stress and thus increases ecosystem C fluxes. However, N enrichment can partially offset the decrease of soil N availability directly by exogenous N supply and indirectly through stimulation on soil N mineralization. Coupled with the relief of soil water limitation and low N demand for plants in late growing season, net N mineralization is relatively high. This conceptual diagram is primarily based on the observation of C and N cycling in this semiarid alpine meadow. For better understanding of these processes, we need long-term monitoring of the seasonal and interannual response of plant productivity and ecosystem CO_2_ fluxes to precipitation pattern and in-depth mechanistic processes (i.e., microbial C and N content as well as its activities and community composition) that drive ecosystem C and N cycling.

## Figures and Tables

**Table 1:** Repeated-measure analyses of variance (ANOVAs) for ecosystem respiration (Reco), aboveground biomass (AGB), soil inorganic N content, and mineralization rate in 2012. *F* and *P* values represent *F* value of ANOVA results and statistical significance, respectively. The significant level is *P* < 0.05.

Model	Reco		AGB		Soil inorganic N content		Soil N mineralization rate	
	*F*	*P*	*F*	*P*	*F*	*P*	*F*	*P*
Warming (W)	8.063	**0.015**	62.290	**<0.001**	0.004	0.948	32.108	**<0.001**
Nitrogen (N)	3.911	0.071	53.157	**<0.001**	30.078	**<0.001**	33.617	**<0.001**
W × N	0.034	0.875	2.710	0.126	3.684	**0.034**	10.375	**<0.001**
Date (D)	31.208	**0.000**	147.502	**<0.001**	60.798	**<0.001**	494.949	**<0.001**
D × W	0.945	0.414	11.565	**<0.001**	15.222	**<0.001**	251.430	**<0.001**
D × N	0.892	0.436	11.300	**<0.001**	13.242	**<0.001**	54.143	**<0.001**
D × W × N	0.268	0.801	4.614	**0.023**	62.886	**<0.001**	46.345	**<0.001**
